# Metagenomics analysis of bacterial structure communities within natural biofilm

**DOI:** 10.1016/j.heliyon.2019.e02271

**Published:** 2019-08-23

**Authors:** Bahaa A. Hemdan, Mohamed Azab El-Liethy, M.E.I. ElMahdy, Gamila E. EL-Taweel

**Affiliations:** a Environmental Microbiology Lab., Water Pollution Research Department, National Research Centre, Dokki, 12622, Giza, Egypt; b Environmental Virology Lab., Water Pollution Research Department, National Research Centre, Dokki, Giza, 12622, Egypt

**Keywords:** Microbiology, Biofilm, NGS analysis, Bacterial lineages, PCR, Public health

## Abstract

The bacterial profiles of natural household biofilm have not been widely investigated. The majorities of these bacterial lineages are not cultivable. Thus, this study aims (i) to enumerate some potential bacterial lineages using culture based method within biofilm samples and confirmed using Biolog GEN III and polymerase chain reaction (PCR). (ii) To investigate the bacterial profiles of communities in two biofilm samples using next generation sequencing (NGS). Forty biofilm samples were cultured and colonies of each selected prevailing potential lineages (*E. coli*, *Salmonella entrica*, *Pseudomonas aeruginosa*, *Staphylococcus aureus* and *Listeria monocytogenes*) were selected for confirmation. From obtained results, the counts of the tested bacterial lineages in kitchen biofilm samples were greater than those in bathroom samples. Precision of PCR was higher than Biolog GEN III to confirm the bacterial isolates. Using NGS analysis, the results revealed that a total of 110,554 operational taxonomic units (OTUs) were obtained for two biofilm samples, representing kitchen and bathroom biofilm samples. The numbers of phyla in the kitchen biofilm sample (35 OTUs) was higher than that in bathroom sample (18 OTUs). A total of 435 genera were observed in the bathroom biofilm sample compared to only 256 in the kitchen sample. Evidences have shown that the empirical gadgets for biofilm investigation are becoming convenient and affordable. Many distinct bacterial lineages observed in biofilm are one of the most significant issues that threaten human health and lead to disease outbreaks.

## 1 Introduction

Naturally, biofilm is composed of different types of microorganisms (*e.g.* bacteria, viruses, fungi) in layer form wrapped by polymer matrices, where bacteria are adherent either on biotic or abiotic surfaces. The adhesion of various types of bacterial lineages could form a biofilm within different aquatic ecosystems. The behaviors of bacterial sessile/biofilm cells differ from the bacterial cells in planktonic state [1]. Whereas approximately 95% of all aquatic microbiomes can adhere onto the inner surfaces of pipe materials and form a biofilm, only 5% are floating in the water column [2]. As well, biofilm is mainly found in both drinking water distribution systems (DWDS) and sink drainage pipes [3, 4]. Although many investigations have been carried out on artificially developed biofilm, the growth and structure of natural biofilm in sink drains remains unknown [5, 6]. Some harmful bacteria can develop and colonize biofilm in the sinks of the kitchen and bathroom for a prolonged period of time [7, 8]. Immune-incompetent people can therefore be subjected to a broad spectrum of opportunistic pathogens in sinks and become more susceptible to infection [9, 10].

The waste of uncooked meat and vegetables, deemed high-risk infectious materials with much more water and food-borne pathogens, passes through the drainage pipe in the kitchen [11, 12]. Likewise, drains of household water are deemed the primary way to disseminate a lot of bacterial lineages around the envirnment [13, 14, 15, 16]. Culture-based method can be regularly used to evaluate the microbial quality of environmental samples [17]. Indeed, the largest of bacterial lineages (more than 99%) in aquatic environmental systems are in a viable but non-culturable (VBNC) state and are thus scarcely detected using culture-based techniques [18, 19]. These cumbersome troubles can, however, be overcome by using culturally independent molecular techniques, including polymerase chain reaction (PCR) and next-generation sequencing (NGS) [7]. Even though the PCR is presently a valuable tool for detecting non-cultivable pathogenic bacteria, it cannot differentiate between healthy and non-viable bacterial cells [20]. To overcome this obstacle, PCR using particular dyes have been using after cultivation of bacteria to provide more information about viability of bacterial community [21, 22].

NGS of bacterial 16S rRNA gene amplicon offers deeper insight into the community composition than clone libraries or fingerprinting technique [23]. Current NGS evaluation involves the sequencing of different variable regions of the bacterial 16S rRNA gene [24, 25]. Classically, the amplicon sequencing of the 16S rRNA gene used in earlier research may identify bacterial populations but is not adequate for recognizing pathogens in a complicated environment including biofilm [26, 28]. The resolution of the commonly utilized 16S rRNA gene may not always be adequate to recognize pathogen, so that more particular target genes are required to identify those organisms [28, 29]. Besides, NGS methods also help to acquire full genetic sequences from uncultivated microorganisms [30, 31]. Earlier NGS studies have also given precious insights and have often been used to analyze DNA from microbial communities in biofilm samples without previous cultivation need [32, 33]. Thereby, this research seeks to enumerate some prospective bacterial lineages using biofilm sample culture-based technique and verified using Biolog GEN III and PCR and to investigate the bacterial profiles communities using next generation sequencing (NGS) for biofilm samples.

## 2 Materials and methods

### 2.1 Biofilm sampling and preparation

Forty natural biofilm samples were randomly collected from bathroom (n = 10) and kitchen (n = 30) sink drainage pipes (30 km south of Cairo, Egypt) under aseptic condition, the inhabitants of household were non-vegetarian. The plastic-based pipes containing biofilm samples were preserved in ice box and immediately transferred to the laboratory within 2 h for microbiological examination according to American Public Health Association (APHA) [34]. The samples were harvested by scraping 10 cm^2^ from the inner surface of pipes using sterile cotton swabs. A small round sampler of an area (10 cm^2^) was found to collect an *average* of 0.45 *g* ± 0.65 wet weight of natural biofilm sample with grey color*.* The swabs were submerged into tubes each containing 10 ml sterile distilled water and homogenized using a vortex agitator for 5 min.

### 2.2 Culture method for enumeration of bacterial biofilm

*E. coli*, *Salmonella entrica*, *Pseudomonas aeruginosa*, *Staphylococcus aureus* and *Listeria monocytogenes* were enumerated in all biofilm samples using the spread plate method according to American Public health Association (APHA) [34]. Samples were appropriately diluted from tenfold serial dilution depending on the cell concentration. To enumerate *E. coli*, 100 μl of suspended biofilm cells were transferred onto ECC agar (HiMedia, India). HiCrome Improved *Salmonella* agar (HiMedia, India) was used to enumerate *Salmonella*, and HiCrome *Listeria* selective agar (HiMedia, India) was used to enumerate *Listeria* biofilm cells. HiCrome Aureus agar (HiMedia, India) was used to enumerate *Staphyoloccus* biofilm cells and HiFluoro *Pseudomonas* agar (HiMedia, India) was used for enumerate *Pseudomonas.* The biofilm formation in all experimental designs is expressed in CFU/cm^2^. Two typical bacterial colonies from each bacterial pathogen were isolated and kept in tryptic soy broth (TSB) with 10% glycerol (BD, Germany) at -40 °C for further identification using PCR and Biolog GEN III [34].

### 2.3 Identification of phenotypic bacterial isolates using Biolog GEN III

The Biolog GEN III system provides a better method to identify a wide spectrum of bacteria. This system can also give phenotyping fingerprint and full picture for bacterial isolates. Typical colonies of each bacterial lineage were picked from the surface of tryptic soy agar (TSA) media to be identified using the Biolog GEN III system (BIOLOG, USA) according to the manufacturer's instructions. A top of single colony was taken using a sterile disposable swab and inoculated into 10 ml of inoculating fluid (IF-A) (Biolog Inc, USA). The inoculated IF-A was dispensed into 96 wells of a microplate (100 μl per well) using a multichannel repeating pipettor. The microplate was incubated at 37 °C for 24h. The reading was carried out automatically by the computerized MicroStation™ system (Biolog Inc, USA) with the fingerprint data which was previously fed into the software (OmniLog® Data Collection) and used to identify the bacteria from their phenotypic patterns in the GEN III MicroPlate [35, 36, 37].

### 2.4 Molecular identification of bacterial isolates using PCR

#### 2.4.1 DNA extraction of bacterial isolates

DNA extraction of bacterial isolates was carried out using a Presto^TM^ Mini gDNA bacterial kit (Geneaid, Taiwan) according to the manufacturer's instructions. The quantity of the extracted DNA was measured by determining absorbance at 260 nm and 280 nm using NanoDrop™ 2000/2000c Spectrophotometers (USA), after which the A260/A280 ratios were calculated with three replicates. The concentration of the extracted DNA was within the acceptable range (1.6–1.8 ng/μL) according to Fontana et al. [38] and Lucena-Aguilar et al. [39].

#### 2.4.2 The PCR amplification and conditions for bacterial biofilm isolates

The confirmation of *E. coli, Salmonella entirca, Pseudomonas areuginosa, Staphylococcus aureus* and *Listeria monocytogenes* isolates were carried out with separate PCR primer as shown in Table 1. The primers used in this study were synthesized by Macrogen Co. (Republic of Korea). Then, PCR was performed in a total volume of 20 μl consisting of 4 μl of 1x FIREPol® Master Mix (Solis BioDyne, Estonia) Ready to use with 12.5 mM MgCl_2_, 0.5 μl of each primer (final concentration, 10 pmol), 12.5 μl of nuclease-free water and 2.5 μl of template DNA. Each PCR assay included a negative and positive control. *E. coli* ATCC 25922, *Salmonella enterica* serovar Typhimurium ATCC 14028, *Staphylococcus aureus* ATCC 25923, *Pseudomonas aeruginosa* ATCC 10145 and *Listeria monocytogenes* ATCC 25152 were used as positive controls for each PCR run. PCR reactions were conducted in a Bio-Rad T100™ thermal cycler with a specific annealing temperature for each set of primers, as shown in Table 1. Subsequently, the amplified products were analyzed via agarose gel electrophoresis. Gels were stained with Ethidium bromide (0.005%, w/v) and visualized under a UV trans-illuminator with a UVP BioDoc-it imaging system.

**Table 1:** Primer sets used for detection of bacterial isolates.

Bacterial strains	Primer Name	Primer sequence (5′ to 3′)	Annealing temp. °C	product size (bp)	References
*E. coli*	URL-301	TGTTACGTCCTGTAGAAAGCCC	55/30 sec.	153	[40]
	URR-432	AAAACTGCCTGGCACAGCAATT			
*Salmonella*	SAL-1F	GTA GAA ATT CCC AGCGGG TAC TG	60/30 sec.	438	[41]
	SAL-2R	GTA TCC ATC TAGCCA ACC ATT GC			
*Pseudomonas*	PA-GS-F	GACGGGTGAGTAATGCCTA	54/20 sec.	610	[42]
	PA-GS-R	CACTGGTGTTCCTTCCTATA			
*Listeria*	S1F	AGT CGG ATAGTA TCC TTA C	60/30 sec.	460	[43]
	S1R	GGC TCT AAC TAC TTG TAG GC			
*Staphylococcus*	*clfA*-F	GCAAAATCCAGCACAACAGGAAACGA	55/30 sec.	638	[44]
	*clfA*-R	CTTGATCTCCAGCCATAATTGGTGG			

For determination of detection limit of all tested bacterial lineages, genomic DNA of fresh 24hr-bacterial culture was extracted and tenfold serial dilution was performed for each bacterial DNA extract. After PCR assay, detection limit of *E. coli* (ATCC 25922), *Salmonella enterica* serovar Typhimurium (ATCC 14028), *Staphylococcus aureus* (ATCC 25923), *Pseudomonas aeruginosa (*ATCC 10145)*,* and *Listeria monocytogenes* (ATCC 25152) was approximately 3 × 10^2^ to 3 × 10^3^ CFU/ml in pure culture.

### 2.5 NGS of biofilm samples

#### 2.5.1 Total DNA extraction

Two biofilm samples were collected from kitchen and bathroom sink drainage pipes. Samples were harvested as described by Hemdan et al. [45]. DNA was then extracted using a MOBIO Power Soil DNA isolation kit (USA) according to the manufacturer's instructions.

#### 2.5.2 Sequencing data preparation

MiSeq standard operating procedure (SOP) was applied for sequencing sample preparations. Briefly, 2μl of the total DNA from each sample was used as a template with primers containing the Illumina adaptor sequence and universal V4 region of 16S rRNA gene, and amplification was done in triplicate using the Maxime PCR PreMix Kit, iNtRON (Republic of Korea). The acquired PCR products were further gel-purified using an AccuPrep Gel Purification kit (Bioneer Inc., Republic of Korea). All obtained DNA were quantified using Qubit (Invitrogen, CA, USA), after which equimolar purified amplicons were pooled and stored at -20 °C until sequenced. Then, amplicons were sequenced using the Illumina MiSeq platform at Macrogen Inc. (Seoul, Republic of Korea) according to the manufacturer's instructions.

### 2.6 Statistical analyses

Statistical analysis was carried out using the software version 5.0 (USA) of GraphPad Prism. A Pearson correlation to clarify the possible correlation in test biofilm samples between the concentrations of explored bacterial lineages and two-way analysis of variance (*ANOVA*) and Student's t-test were performed to evaluate significance (*P* < 0.05) between the source of biofilm samples (kitchen and bathroom).

## 3 Results and discussion

### 3.1 Enumeration and isolation of bacterial lineages from biofilm

Water and drainage pipes contain different microbial communities, including bacteria, viruses, and fungi, especially in biofilm formation. Besides, showerhead and sink drains biofilm plays a major role as one of the possible sources of infection [46, 47]. To explore the bacterial communities in natural biofilm, a culture-dependent method was used to detect five potential bacterial pathogens including *E. coli*, *Salmonella enterica*, *Pseudomonas aeruginosa*, *Staphylococcus aureus* and *Listeria monocytogenes*. To confirm the culture based characterization, the isolates were further identified using PCR and Biolog GEN III.

**Fig. 1:**
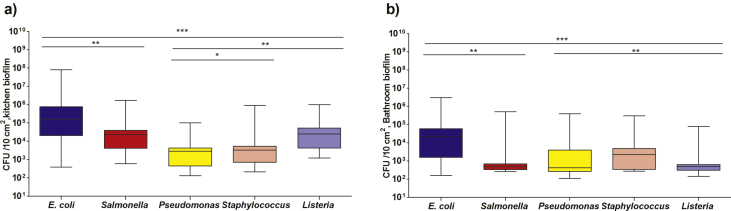
Average counts of some bacterial pathogens in household biofilm samples, ∗ indicated to low correlation (*P* ≤ 0.05), ∗∗ indicated to moderate correlation (*P* ≤ 0.01), ∗∗∗ indicated to high correlation (*P* ≤ 0.001).

Colony forming units of *E. coli* numbers in kitchen biofilm samples (3.8 × 10^2^ to 8.0 × 10^7^ CFU/10 cm^2^) were higher than those in bathroom biofilm samples (1.6 × 10^2^ to 6.2 × 10^4^ CFU/10 cm^2^). Furthermore, *E. coli* counts were the highest in all biofilm samples among the tested biofilm pathogens (Fig. 1). Few studies have shown the confirmation of *E. coli* biofilm in the water networks [48, 49]. Total numbers of *Salmonella enterica* cells varied from 5.9 × 10^2^ to 1.7 × 10^6^ CFU/10 cm^2^. The highest *Salmonella enterica* counts were observed in kitchen drainage pipe biofilm samples (Fig. 1). Statistically, results found up that there are good correlations with high significance between tested bacteria lineages in biofilm. These results are Comparable with those reported by Hemdan et al. [50], who explained that pathogens are present in biofilm samples because of their capability to form biofilm on different surfaces under different environmental conditions, and these pathogens may act as a potential sources of food borne bacterial illness. *Salmonella* can form biofilm on plastics [51]. Moreover, bacterial pathogens can be found in water supplies due to their ability to colonize surfaces and replicate in biofilm of distribution system pipes and other microhabitats. Meanwhile, pipes that transport drinking water through municipal drinking water distribution systems (DWDS) are challenging habitats for microorganisms. Distribution networks are dark, oligotrophic and contain disinfectants; yet microbes frequently form biofilm attached to interior surfaces of DWDS pipes [52, 53].

The results shown in Fig. 1 (a, b) indicate that the total numbers of *Listeria*, *Staphylococcus aureus* and *Pseudomonas aeruginosa* cells varied from 1.2 × 10^3^ to 5.3 × 10^5^, 2.1 × 10^2^ to 9.2 × 10^5^ and 1.3 × 10^2^ to 1.0 × 10^5^ CFU/10 cm^2^ in kitchen biofilm samples, respectively. While in the bathroom samples, they were 2.5 × 10^2^ to 7.2 × 10^2^, 2.7 × 10^2^ to 3.5 × 10^3^ and 1.1 × 10^2^ to 5.7 × 10^3^ CFU/10 cm^2^, respectively. These results may be due to the washing of contaminated vegetables and fruits that were irrigated by insufficiently treated wastewater, which may be a source of non-pathogenic and pathogenic microbes. Several studies have identified sink drains in households as possible cause of outbreaks [50, 54]. In addition, the kitchens and bathrooms of households have been found to contribute to the transmission of pathogenic bacteria [55]. Many microbes can cause infections at low doses since they can survive from several hours to weeks on the moist surfaces of kitchens and bathrooms [56]. The minimal infectious doses for *E. coli* and *Salmonella* were ranged from 10^6^ to 10^8^ and 10^4^ to 10^7^ cells, respectively while for *Staphylococcus aureus* and *Listeria monocytogenes* were ranged from 10^3^ to 10^8^ and from 10 to 10^8^ cells in healthy people [57, 58, 59]. Moreover, *Pseudomonas aeruginosa* which considered as an opportunistic pathogen and is able to colonize healthy people without disease, their infectious dose is still unknown [60, 61]. These harbor pathogens, originating from various sources such as infected individuals, unclean food and inhaled contaminated water, could always be distributed and transmitted in various ways, such as food manufacturing and regular contact with heavy-density surfaces of pathogenic bacteria [62].

In parallel with identification using the Biolog system, identification using the molecular PCR method with genus- and lineages -specific oligonucleotide primers was performed according to Sandle et al. [63] and Chojniak et al. [64]. As shown in Table 2, the accuracy percentages of all confirmed isolates via Biolog were lower than those of PCR. Thus, PCR is more accurate for the confirmation of bacterial isolates than the phenotypic method (Biolog GEN III). This improved efficacy may occur because PCR is able to detect the nucleic acids of bacteria, while Biolog GEN III depends on their metabolic activities. The DNA-based methods are superior to conventional automated phenotypic systems [27, 28, 38].

**Table 2:** Number and percentage of bacterial biofilm isolates isolated from different sink drainage pipes confirmed by Biolog GEN III and PCR.

Biofilm Sample	*E. coli*				*Salmonella*				*Pseudomonas*				*Staphylococcus*				*Listeria*			
	Biolog		PCR		Biolog		PCR		Biolog		PCR		Biolog		PCR		Biolog		PCR	
	+	%	+	%	+	%	+	%	+	%	+	%	+	%	+	%	+	%	+	%
Kitchen	37	61.6	49	81.6	43	71.6	52	86.6	48	80	58	96.6	44	73.3	53	88.3	39	65	51	85
Bathroom	16	80	18	90	13	65	20	100	10	70	20	100	13	65	19	95	14	70	18	90

**Fig. 2:**
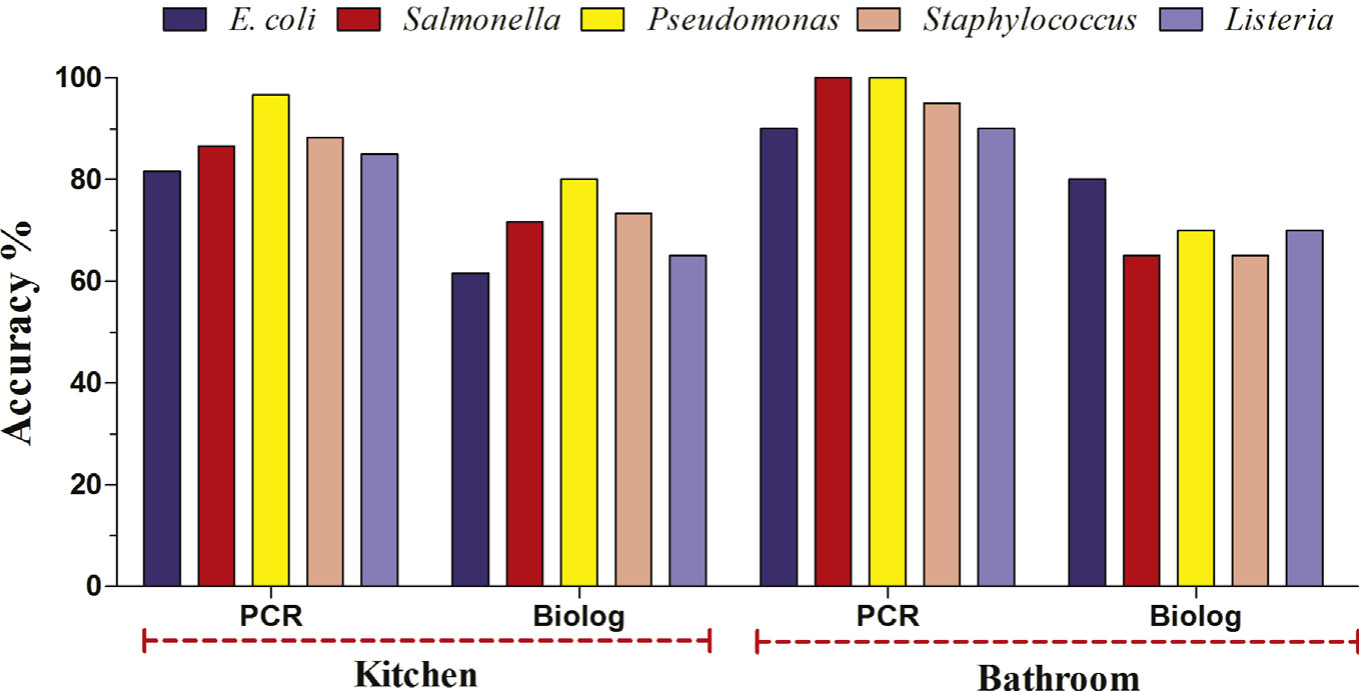
Accuracy percentages of bacterial isolates confirmation via Biolog GEN III system and PCR from biofilm collected from kitchen (a) and bathroom drains (b).

The findings are graphically illustrated in Fig. 2. In terms of the confirmation of kitchen biofilm bacterial isolates, the accuracy percentages of *E. coli*, *Salmonella entrica*, *Pseudomonas aeruginosa*, *Staphylococcus aureus* and *Listeria monocytogenes* using Biolog GEN III were 61.6, 71.6, 65, 73.3 and 80%, respectively. Since the percentages for PCR were higher than those of the Biolog GEN III results in Fig. 2, it can be concluded that the usage of PCR for bacterial confirmation in biofilm samples is preferable. Furthermore, PCR is a promising method for detecting and confirming the pathogens originating in biofilm due to its accuracy related to difficult-to-identify isolates [65]. In contrast, the conventional phenotypic systems require a prolonged cultivation period for the suspected bacteria and pure bacterial cultures for different biochemical assays [66]. One key to the effective use of such systems is the ability to draw upon databases that can be augmented with new data gleaned from atypical or novel isolates [35].

### 3.2 Next generation sequencing (NGS)

**Fig. 3:**
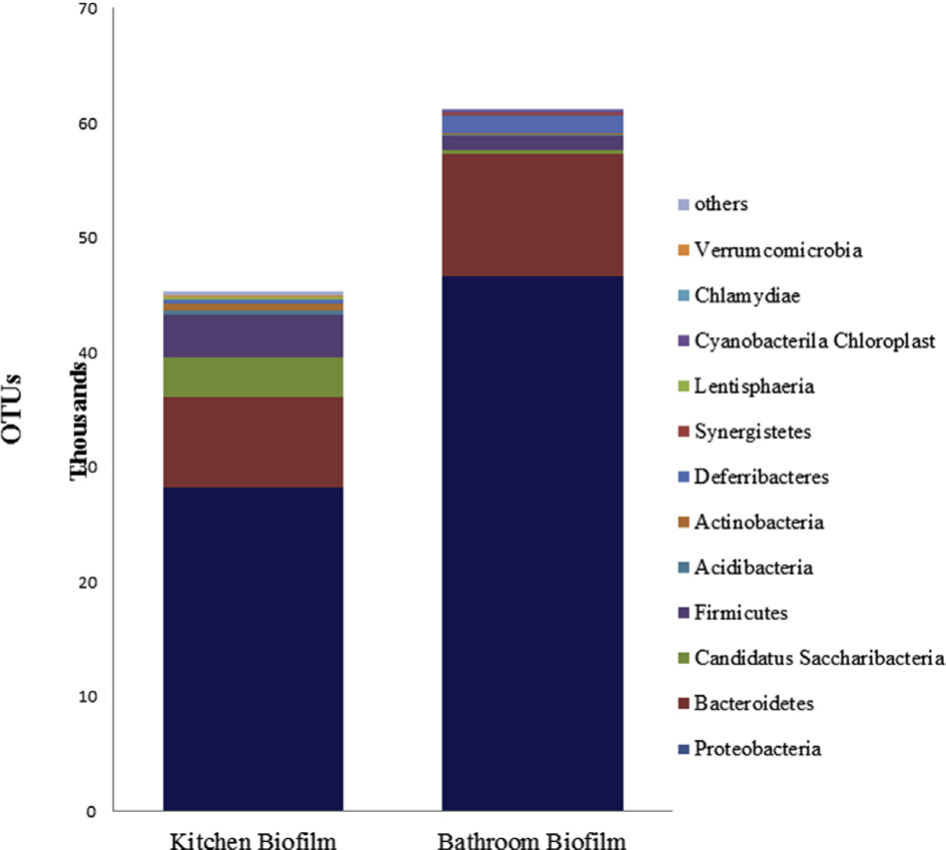
Relative taxonomic distribution of different bacterial phylogenetic groups in biofilm collected from kitchen and bathroom drainage pipes. Analysis of 16S rRNA gene sequences was done in comparison with the RDP database.

The present study clarify a profile of the bacterial community structure of natural biofilm by conducting a taxonomic analysis using NGS based on Ribosomal Database Project (RDP) Classifier with read length of >250 bp identified sequences. The results revealed that the *Proteobacteria* had the highest relative abundance of OTUs on the two natural biofilm samples. Through a comparison of kitchen and bathroom biofilm samples, the results show that the OTUs for the kitchen biofilm had lower relative abundance than those of bathroom biofilm (62% and 75%, respectively). In addition, the relative abundance of OTUs for *Bacteroidetes* in kitchen biofilm (18%) was lower than that in bathroom biofilm (19%). Moreover, the phyla *Candidatus Saccharibacteria* and *Firmicutes* had the highest relative abundances of OTUs (8%) in kitchen biofilm. The data presented in Fig. 3 reveal that the variation of bacterial structure community for both kitchen and bathroom biofilm is very large. Furthermore, the bacterial community structure differed between kitchen and bathroom biofilm.

In contrary, Schmeisser et al. [67], Simões et al. [68] found that Betaproteobacteria was the most dominant class in their samples and this may be due to various reasons. The source of pathogenic microorganisms was different in the two studied biofilm. While the main source of microorganisms in kitchens that can form biofilm is the water resulting from uncooked meat and vegetable waste [11, 12].

The main source of bathroom microorganisms is the water resulting from domestic hand and face washing. Furthermore, the differences in findings may be attributed to the differences in the growth stages and ages of the studied biofilm. These results support other studies that have reported the development of a greater biofilm biomass on metals than on plastics. Furthermore, NGS analysis provides an effective supplementary tool in taxonomic analysis based on 16S rRNA genes. Moreover, NGS has helped microbiologists reveal the genome of the rest of the 99% of non-cultivable microbes, which enables a better understanding of global microbial ecology and has helped meet the current demand for novel enzymes [69].

**Fig. 4:**
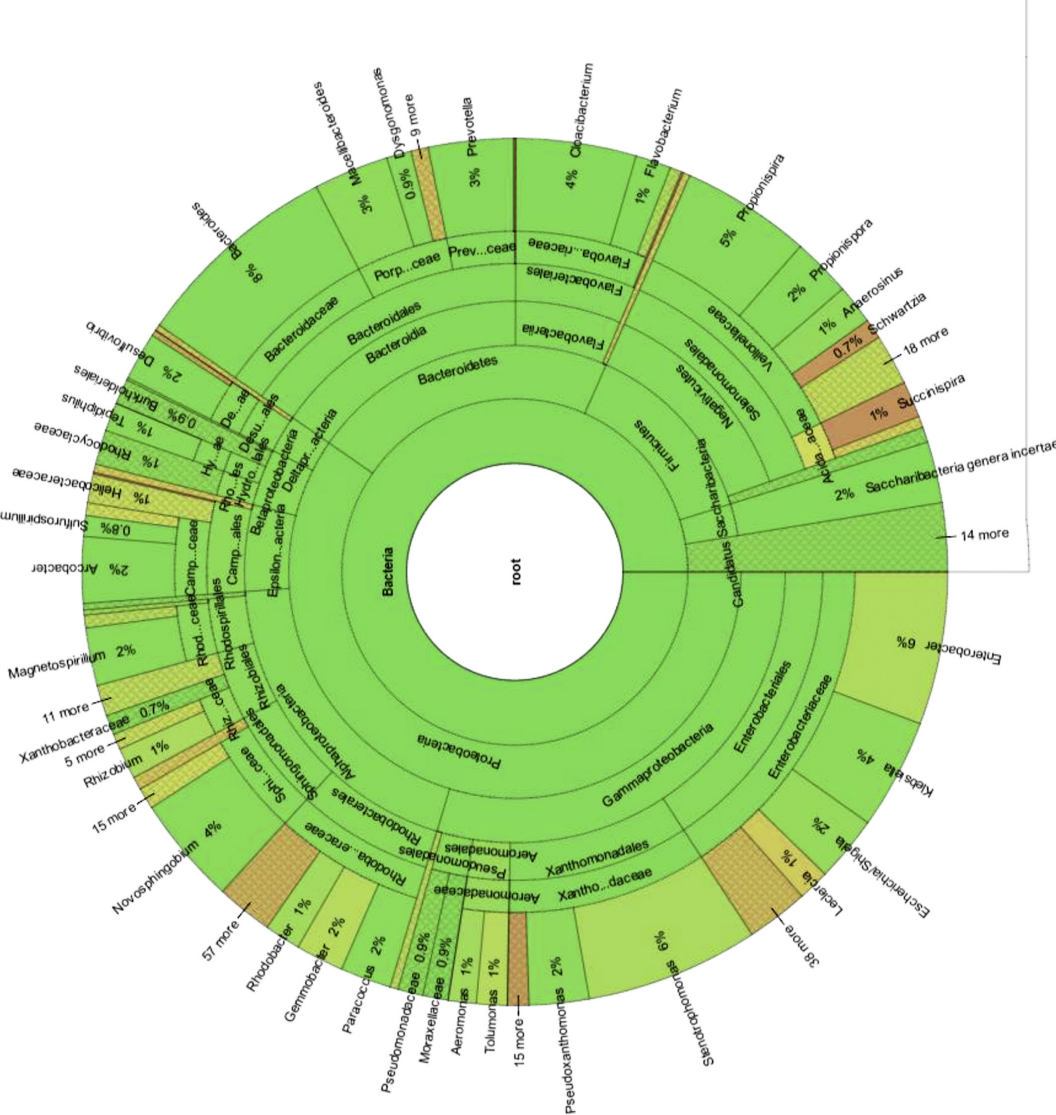
Hierarchical tree representing taxonomic relationships of most abundance bacterial community structure of kitchen biofilm classified by RDP Classifier.

Based on the taxonomic results, Proteobacteria, particularly Alphaproteobacteria, were dominant in the natural biofilm collected from kitchen (Fig. 4). This finding agrees with other studies, indicating that biofilm harboring opportunistic pathogens are common issues [70, 71]. Furthermore, these findings are well matched with those reported by Chao et al*.* [72], who found that the relative abundance of Proteobacteria was larger than that of other phyla. These proteobacteria include a wide range of pathogens such as *Escherichia*, *Vibrio* and *Salmonella* and many other common genera [73]. However, other studies have reported contrary results, with Betaproteobacteria, rather than Alphaproteobacteria, as the most dominant class in their biofilm, which might be attributable to several explanations, including differences among applied molecular methods.

At the family level for Bacteroidetes, Flavobacteriaceae (2809 OTUs), Bacteroidaceae (1917 OTUs) and Porphyromonadaceae (1529 OTUs) were the most dominant families present (of all sequences, as shown in Fig. 4). At the genus level, there was no significant difference in bacterial communities in the kitchen biofilm samples - *Cloacibacterium* (2142 OTUs), *Bacteroides* (1917 OTUs), and *Prevotella* (1167 OTUs).

At the family level for Firmicutes, significant differences were observed in the bacterial communities. Veillonellaceae (2925 OTUs), Acidaminococcaceae (142 OTUs), Lachnospiraceae (52 OTUs), and Clostridiaceae 1 (35 OTUs) were the most dominant families present. At the genus level, there was no significant difference in bacterial communities in the kitchen biofilm samples - *Propionispira* (1256 OTUs), *Propionispora* (581 OTUs), and *Anaerosinus* (343 OTUs). At the genus level, there was a significant difference in the bacterial communities in the kitchen biofilm samples. *Pseudoxanthomonas* (1923), *Novosphingobium* (2458 OTUs), *Klebsiella* (712 OTUs), *Arcobacter* (759 OTUs), *Desulfovibrio* (758 OTUs), *Escherichia/Shigella* (396 OTUs), *Enterobacter* (395 OTUs), *Rhodobacter (*365 OTUs), and *Pseudomonas* (156 OTUs) were the nine major genera present in the kitchen samples (of all sequences, as shown in Fig. 4).

The numbers of phyla in the kitchen and bathroom biofilm samples were 19 and 18, respectively. Despite the low variance in the identified number of bacterial phyla and classes found in the two biofilm sampling sites, the number of OTUs at the family level in kitchen biofilm samples was 117 compared to 95 in bathroom samples. Despite the kitchen biofilm having the highest family numbers, the number of genera was only 256. Moreover, the number of OTUs at the genus level in bathroom biofilm was 435 (Table 3).

**Table 3:** Hierarchy classification in order with counts in kitchen (a) bathroom (b) household biofilm.

Type of sample	Hierarchy classification					
	Domain	Phylum	Class	Order	Family	Genus
Kitchen Biofilm	1	19	35	63	117	256
Bathroom Biofilm	1	18	37	56	95	435

**Fig. 5:**
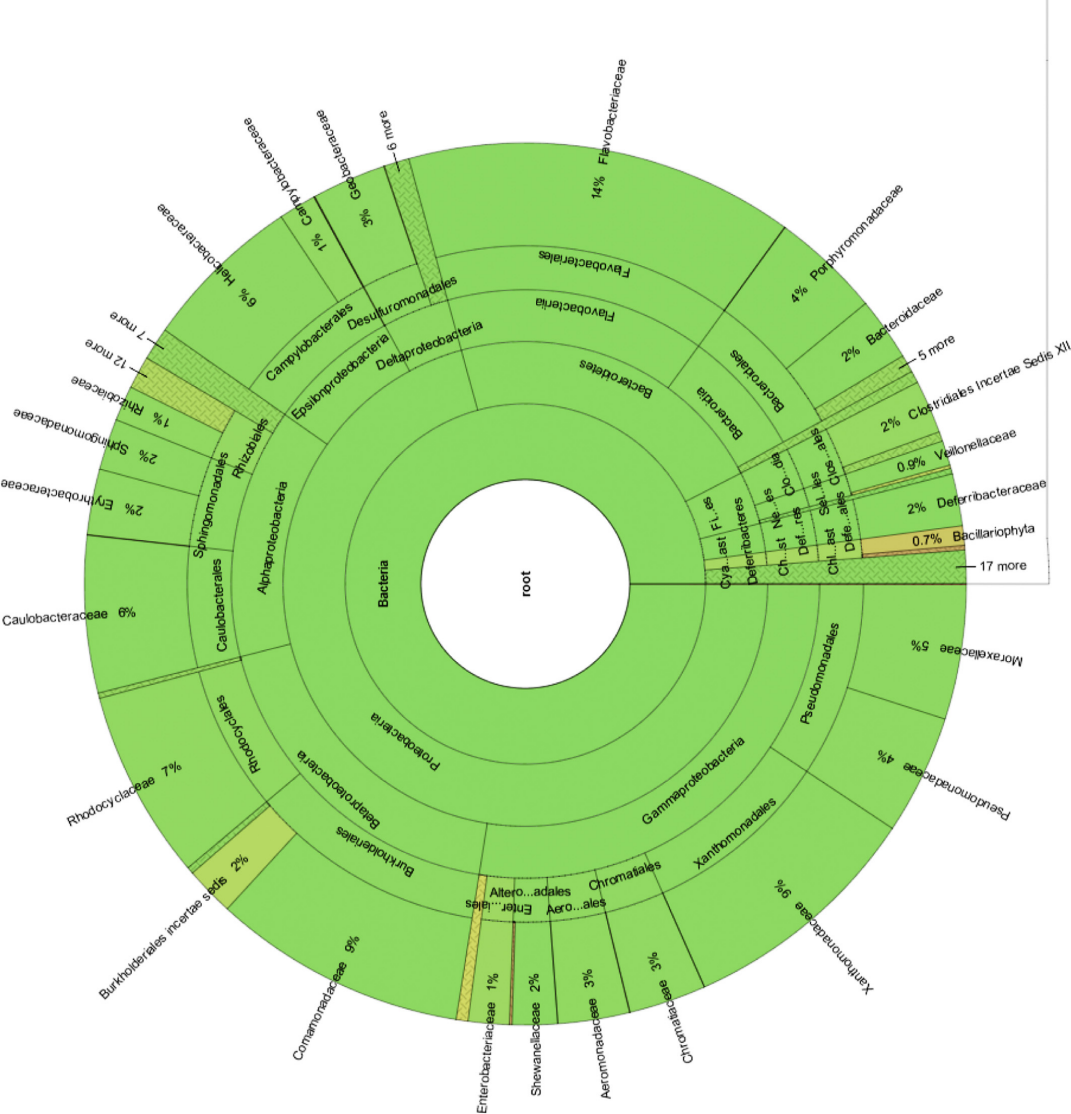
Hierarchical tree representing taxonomic relationships of most abundance bacterial community structure of bathroom biofilm classified by RDP Classifier.

At the family level for Proteobacteria, significant differences were observed in bacterial communities (*P* < 0.01). Xanthomonadaceae (7541 OTUs), Caulobacteraceae (5246 OTUs), Rhodocyclaceae (4994 OTUs), Comamonadaceae (4552 reads), Erythrobacteraceae (3838 OTUs), Helicobacteraceae (2722 OTUs), Chromatiaceae (1659 OTUs), Geobacteraceae (1636 OTUs), Sphingomonadaceae (1587 OTUs), Pseudomonadaceae (1383 OTUs), and Moraxellaceae (1323 OTUs) of all sequences, as shown in Fig. 5, were the most dominant families detected. At the genus level, there was a significant difference in bacterial communities in the bathroom biofilm samples. *Sulfuricurvum* (2713 OTUs), *Aquabacterium* (553 OTUs), *Azospira* (47,836 OTUs), *Bosea* (514 OTUs), *Porphyrobacter* (3317 OTUs), *Brevundimonas* (1392 OTUs), *Pseudoxanthomonas* (5776 OTUs), *Rheinheimera* (1659 OTUs), *Acinetobacter* (1227 OTUs), *Pseudomonas* (1048 OTUs), and *Geobacter* (1620 OTUs) were the eleven major genera present in the bathroom biofilm samples (of all sequences, Fig. 5).

At the family level for Bacteroidetes, Flavobacteriaceae (7597 OTUs) was the most dominant family present, followed by Porphyromonadaceae (1727 OTUs). At the genus level, there was no significant difference in bacterial communities in the biofilm of bathroom samples. *Cloacibacterium* (6948 OTUs) and *Macellibacteroides* (1308 OTUs) were the two major genera present in the bathroom biofilm samples. At the family level for Firmicutes, significant differences were observed in bacterial communities. Clostridiales_IncertaeSedis XII (516 OTUs), followed by Veillonellaceae (276 OTUs), were the most dominant families present (of all sequences, Fig. 5). At the genus level, there was no significant difference in bacterial communities in the bathroom biofilm samples – *Fusibacter* (516 OTUs) and *Propionispira* (85 OTUs). Accordingly, NGS has the potential to be used for routine environmental monitoring.

In environmental monitoring, NGS technologies are of great interest [74] and have been used to realize phylogenetic and NGS analyses [75]. NGS has also been used to both improve the barcoding approach [76] and to estimate biodiversity, especially in fresh water [77]. However, biofilm of domestic drains are well known to harbor large numbers of different microbial communities. Many of the microbes identified in the present study are related to typical natural biofilm, and their presence in biofilm has been described in many studies. The bacterial communities present in two samples from kitchen and bathroom biofilm were analyzed using amplicon NGS of the V1–V2 and V3 regions of the 16S rRNA gene. Based on the NGS results, Proteobacteria, in particular Alphaproteobacteria, were dominant in the biofilm of the kitchen domestic drains, followed by Gammaproteobacteria. In contrast, in the biofilm of bathroom domestic drains, Gammaproteobacteria were dominant, followed by Alphaproteobacteria. In addition, the findings of the present study are similar to the analytical results of several previous studies [70, 71] that also observed Alphaproteobacteria as dominant in biofilm.

## 4 Conclusion

Getting assumed that microbiological populations could vary between biofilm samples obtained from kitchen and bathroom drains. Although cultivation techniques are popularly used, they do not detect the entire spectrum of particularly uncultivable bacteria. Data shows that the bacterial isolates are being confirmed using both PCR and Biology. Precision of PCR in confirmation of the bacterial isolates is higher than Biolog. As well, results of NGS also are given better information about most prevalent phyla, family, genera and lineage numbers of bacteria. The results of this research illustrate that *Proteobacteria* are the largest relative abundance on the two natural biofilm samples. From the obtained results, it can be concluded that the densities of tested bacterial strains presented in biofilm of kitchen drains were greater than biofilm existing in bathroom drains. In spite of small sample size, the current investigation provides good information about presence of various types of lineages in biofilm development especially in domestic drain conduits, further studies must be performed in near future.

## Declarations

### Author contribution statement

Bahaa A. Hemdan: Conceived and designed the experiments; Performed the experiments; Contributed reagents, materials, analysis tools or data; Wrote the paper.

Mohamed Azab El-Liethy: Conceived and designed the experiments; Analyzed and interpreted the data; Contributed reagents, materials, analysis tools or data; Wrote the paper.

M.E.I. ElMahdy: Conceived and designed the experiments; Contributed reagents, materials, analysis tools or data; Wrote the paper.

Gamila E. EL-Taweel: Contributed reagents, materials, analysis tools or data; Wrote the paper.

### Funding statement

This work was supported by the Science and Technology Development Fund (STDF) - grant Number 15031.

### Competing interest statement

The authors declare no conflict of interest.

### Additional information

No additional information is available for this paper.

## References

[1] Besemer K. (2015). Biodiversity, community structure and function of biofilms in stream ecosystems. Res. Microbiol..

[2] Liu S., Hou Y., Chen X., Gao Y., Li H., Sun S. (2014). Combination of fluconazole with non-antifungal agents: a promising approach to cope with resistant *Candida albicans* infections and insight into new antifungal agent discovery. Int. J. Antimicrob. Agents.

[3] Wingender J., Flemming H.C. (2004). Contamination potential of drinking water distribution network biofilms. Water Sci. Technol..

[4] Wingender J., Flemming H.C. (2011). Biofilms in drinking water and their role as reservoir for pathogens. Int. J. Hyg Environ. Health.

[5] Giao M.S., Azevedo N.F., Wilks S.A., Vieira M.J., Keevil C.W. (2008). Persistence of *Helicobacter pylori* in heterotrophic drinking-water biofilms. Appl. Environ. Microbiol..

[6] Moritz M.M., Flemming H.-C., Wingender J. (2010). Integration of *pseudomonas aeruginosa* and *Legionella pneumophila* in drinking water biofilms grown on domestic plumbing materials. Int. J. Hyg Environ. Health.

[7] Douterelo I., Boxall J.B., Deines P., Sekar R., Fish K.E., Biggs C.A. (2014). Methodological approaches for studying the microbial ecology of drinking water distribution systems. Water Res..

[8] Douterelo I., Jackson M., Solomon C., Boxall J. (2017). Spatial and temporal analogies in microbial communities in natural drinking water biofilms. Sci. Total Environ..

[9] Sinclair R.G., Gerba C.P. (2011). Microbial contamination in kitchens and bathrooms of rural Cambodian village households. Lett. Appl. Microbiol..

[10] Flemming H., Wingender J., Szewzyk U., Steinberg P., Rice S.A., Kjelleberg S. (2016). Biofilms: an emergent form of bacterial life. Nat. Rev. Microbiol..

[11] Francis G.A., Thomas C., O’Beirne D. (1999). The microbiological safety of minimally processed vegetables. Int. J. Food Microbiol..

[12] Berger C.N., Sodha S.V., Shaw R.K., Griffin P.M., Pink D., Hand P., Frankel G. (2010). Fresh fruit and vegetables as vehicles for the transmission of human pathogens. Environ. Microbiol..

[13] Cogan T.A., Bloomfield S.F., Humphrey T.J. (1999). The effectiveness of hygiene procedures for prevention of cross-contamination from chicken carcases in the domestic kitchen. Lett. Appl. Microbiol..

[14] Bloomfield S.F. (2001). Gastrointestinal disease in the domestic setting. What are the issues?. J. Infect..

[15] McBain A.J., Gilbert P. (2001). Biocide tolerance and the harbingers of doom. Int. Biodeterior. Biodegrad..

[16] Cogan T.A., Slader J., Bloomfield S.F., Humphrey T.J. (2002). Achieving hygiene in the domestic kitchen: the effectiveness of commonly used cleaning products. J. Appl. Microbiol..

[17] Swan J.S., Deasy E.C., Boyle M.A., Russell R.J., O’Donnell M.J., Coleman D.C. (2016). Elimination of biofilm and microbial contamination reservoirs in hospital washbasin U-bends by automated cleaning and disinfection with electrochemically activated solutions. J. Hosp. Infect..

[18] Oliver J.D. (2005). The viable but nonculturable state in bacteria. J. Microbiol..

[19] Lautenschlager K., Boon N., Wang Y., Egli T., Hammes F. (2010). Overnight stagnation of drinking water in household taps induces microbial growth and changes in community composition. Water Res..

[20] Pathak E., El-Borai F.E., Campos-Herrera R., Jonhson E.G., Stuart R.J., Graham J.H., Duncan L.W. (2012). Use of real-time PCR to discriminate predatory and saprophagous behavior by nematophagous fungi. Fungal Biol.

[21] Nocker A., Sossa-Fernandez P., Burr M.D., Camper A.K. (2007). Use of propidium monoazide for live/dead distinction in microbial ecology. Appl. Environ. Microbiol..

[22] Henne K., Kahlisch L., Brettar I., Höfle M.G. (2012). Analysis of structure and composition of bacterial core communities in mature drinking water biofilms and bulk water of a Citywide Network in Germany. Appl. Environ. Microbiol..

[23] Keshri J., Pradeep A.S., Sime-Ngando T. (2017). Distinctive patterns in the taxonomical resolution of bacterioplankton in the sediment and pore waters of contrasted freshwater lakes. Microb. Ecol..

[24] Goodrich J.K., Di Rienzi S.C., Poole A.C., Koren O., Walters W.A., Caporaso J.G., Knight R., Ley E.E. (2014). Conducting a microbiome study. Cell.

[25] D’Amore R., Ijaz U.Z., Schirmer M., Kenny J.G., Gregory R., Darby A.C., Shakya M., Podar M., Quince C., Hall N. (2016). A comprehensive benchmarking study of protocols and sequencing platforms for 16S rRNA community profiling. BMC Genomics.

[26] Ashelford K.E., Chuzhanova N.A., Fry J.C., Jones A.J., Weightman A.J. (2005). At least 1 in 20 16S rRNA sequence records currently held in public repositories is estimated to contain substantial anomalies. Appl. Environ. Microbiol..

[27] Bosshard P.P., Zbinden R., Abels S., Boddinghaus B., Altwegg M., Bottger E.C. (2006). 16S rRNA gene sequencing versus the API 20 NE system and the Vitek 2 ID-GNB card for identification of nonfermenting gram- negative bacteria in the clinical laboratory. J. Clin. Microbiol..

[28] Janda J.M., Abbott S.L. (2007). 16S rRNA gene sequencing for bacterial identification in the diagnostic laboratory: pluses, perils, and pitfalls. J. Clin. Microbiol..

[29] Simon C., Danie R. (2011). Metagenomic analyses: past and future trends. Appl. Environ. Microbiol..

[30] Meli K., Kamika I., Keshri J., Momba M.N.B. (2016). The impact of zinc oxide nanoparticles on the bacterial microbiome of activated sludge systems. Sci. Rep..

[31] Garza D.R., Dutilh B.E. (2015). From cultured to uncultured genome sequesces: metagenomics and modeling microbila ecosystems. Cell. Mol. Life Sci..

[32] Delafont V., Brouke A., Bouchon D., Moulin L., Hechard Y. (2013). Microbiome of free-living amoebae isolated from drinking water. Water Res..

[33] Douterelo I., Jackson M., Solomon C., Boxall J. (2016). Microbial analysis of in situ biofilm formation in drinking water distribution systems: implications for monitoring and control of drinking water quality. Appl. Microbiol. Biotechnol..

[34] American Public health Association (APHA) (2012). Standard Methods for the Examination of Water and Wastewater.

[35] Wragg P., Randall L., Whatmore A.M. (2014). Comparison of Biolog GEN III MicroStation semi-automated bacterial identification system with matrix-assisted laser desorption ionization-time of flightmass spectrometry and 16S ribosomal RNA gene sequencing for the identification of bacteria of veterinary interest. J. Microbiol. Methods.

[36] Al-Dhabaan F.A., Bakhali A.H. (2017). Analysis of the bacterial strains using Biolog plates in the contaminated soil from Riyadh community. Saudi J. Biol. Sci..

[37] El-Liethy M.A., Hemdan B.A. (2018). El-Taweel GE Phenotyping using semi-automated BIOLOG and conventional PCR for identification of *Bacillus* isolated from biofilm of sink drainage pipes. Acta Ecol. Sin..

[38] Fontana C., Favaro M., Pelliccioni M., Pistoia E.S., Favalli C. (2005). Use of the MicoSEQ 500 16S rRNA gene-based sequencing for identification of bacterial isolates that commercial automated systems failed to identify correctly. J. Clin. Microbiol..

[39] Lucena-Aguilar G., Sanchez-Lopez A.M., Barberan-Aceituno C., Carrillo-Avila J.A., Lopez-Guerrero J.A., Aguilar-Quesada R. (2016). DNA Source selection for downstream spplications based on DNA quality indicators analysis. Biopreserv. Biobank.

[40] Bej A.K., DiCesare J.L., Haff L., Atlas R.M. (1991). Detection of *Escherichia coli* and *Shigella* spp. in water by using the polymerase chain reaction and gene probes for uid. Appl. Environ. Microbiol..

[41] Aabo S., Rasmussen O.F., Rossen L., Sorensen P.D., Olsen J.E. (1993). *Salmonella* identification by the polymerase chain reaction. Mol. Cell. Probes.

[42] Spilker T., Coenye T., Vandamme P., LiPuma J.J. (2004). PCR based assay for differentiation of *Pseudomonas aeruginosa* from other *Pseudomonas* lineages recovered from cystic fibrosis patients. J. Clin. Microbiol..

[43] Paillard D., Dubois V., Duran R., Nathier F., Guittet C., Caumette P., Quentin C. (2003). Rapid identification of Listeria lineages by using restriction fragment length polymorphism of PCR Amplified 23S rRNA gene fragments. Appl. Environ. Microbiol..

[44] Smeltzer M.S., Gillaspy A.F., Pratt F.L., Thames M.D., Iandolo J.J. (1997). Prevalence and chromosomal map location of *Staphylococcus aureus* adhesin genes. Gene.

[45] Hemdan B.A., El-liethy M.A., Shaban A.M., El-Taweel G.E. (2017). Quantification of the metabolic activities of natural biofilm of different microenvironments. J Environ Sci Technol.

[46] Falkinham J.O., Iseman M.D., de Haas P., van Soolingen D. (2008). *Mycobacterium avium* in a shower linked to pulmonary disease. J. Water Health.

[47] Armbruster C.R., Forster T.S., Donlan R.M., O'Connell H.A., Shams A.M., Williams M.M. (2012). A biofilm model developed to investigate survival and disinfection of *Mycobacterium mucogenicum* in potable water. Biofouling.

[48] Juhna T., Birzniece D., Rubulis J. (2007). (Effect of phosphorus on survival of *Escherichia coli* in drinking water biofilms. Appl. Environ. Microbiol..

[49] Maes S., Vackier T., Nguyen Huu S., Heyndrickx M., Steenackers H., Sampers I., Raes K., Verplaetse A., De Reu K. (2019). Occurrence and characterisation of biofilms in drinking water systems of broiler houses. BMC Microbiol..

[50] Hemdan B.A., El-Liethy M.A., Eissa A.H., Kamel M.M., El-Taweel G.E. (2016). Effect of corroded and non corroded pipe materials on biofilm formation in water distribution systems. World Appl. Sci. J..

[51] Momba M.N., Kaleni P. (2002). Regrowth and survival of indicator microorganisms on the surfaces of household containers used for the storage of drinking water in rural communities of South Africa. Water Res..

[52] Kelly J.J., Minalt N., Culotti A., Pryor M., Packman A. (2014). Temporal variations in the abundance and composition of biofilm communities colonizing drinking water distribution pipes. PLoS One.

[53] Fish K.E., Osbornb A.M., Boxall J. (2016). Characterizing and understanding the impact of microbial biofilms and the extracellular polymeric substance (EPS) matrix in drinking water distribution systems. Environ. Sci. Water Res. Technol..

[54] Blom K. (2015). Drainage systems, an occluded source of sanitation related outbreaks. Arch. Public Health.

[55] Reynolds K.A., Watt P.M., Boone S.A. (2005). Gerba CP Occurrence of bacteria and biochemical markers on public surfaces. Int. J. Environ. Health Res..

[56] Sinclair R.G., Choi C.Y., Riley M.R., Gerba C.P. (2008). Pathogen surveillance through monitoring of sewer systems. Adv. Appl. Microbiol..

[57] Farber J.M., Ross W.H., Harwig J. (1996). Health risk assessment of *Listeria monocytogenes* in Canada. Int. J. Food Microbiol..

[58] Schmid-Hempel P., Frank S.A. (2007). Pathogenesis, virulence, and infective dose. PLoS Pathog..

[59] Bitton G. (2011). Wastewater Microbiology.

[60] Banerjee A., Dangar T.K. (1995). *Pseudomonas aeruginosa*, a facultative pathogen of red palm weevil, Rhynchophorus ferrugineus. World J. Microbiol. Biotechnol..

[61] Pollack M., Mandell G.L., Bennett J.E., Dolin R. (2000). Pseudomonas aeruginosa. Principles and Practice of Infectious Diseases.

[62] Medrano-Felix A., Martinez C., Castro-del Campo N., Leon-Felix J., Peraza-Garay F., Gerba C.P., Chaidez C. (2011). Impact of prescribed cleaning and disinfectant use on microbial contamination in the home. J. Appl. Microbiol..

[63] Sandle T., Skinner K., Sandle J., Gebala B., Kothandaraman P. (2013). Evaluation of the GEN III OmniLog® ID System microbial identification system for the profiling of cleanroom bacteria. Eur. J. Parenter. Pharm. Sci..

[64] Chojniak J., Jałowiecki T., Dorgeloh E., Hegedusova B., Ejhed H., Magnér J., Płaza G. (2015). Application of the BIOLOG system for characterization of *Serratia marcescens* isolated from onsite wastewater technology (OSWT). Acta Biochim. Pol..

[65] Moraes P.M., Perin L.M., Júnior A.S., Nero L.A. (2013). Comparison of phenotypic and molecular tests to identify lactic acid bacteria. Braz. J. Microbiol..

[66] Järvinen A., Laakso S., Piiparinen P., Aittakorpi A., Lindfors M., Huopaniemi L., Piiparinen H., Mäki M. (2009). Rapid identification of bacterial pathogens using a PCR- and microarray based assay. BMC Microbiol..

[67] Schmeisser C., Stockigt C., Raasch C., Wingender C., Timmis K.N., Wenderoth D.F., Flemming H.-C., Liesegang H., Scmitz R.A., Jaeger K.-E., Streit W.R. (2003). Metagenome survey of biofilms in drinking-water networks. Appl. Environ. Microbiol..

[68] Simões L.C., Azevedo N., Pacheco A., Keevil C.W., Vieira M.J. (2006). Drinking water biofilm assessment of total and culturable bacteria under different operating conditions. Biofouling.

[69] Schmeisser C., Steele H., Streit W.R. (2007). Metagenomics, biotechnology with non-culturable microbes. Appl. Microbiol. Biotechnol..

[70] Jang H.J., Choi Y.J., JO Ka (2011). Effects of diverse water pipe materials on bacterial communities and water quality in the annular reactor. J. Microbiol. Biotechnol..

[71] Liu R., Yu Z., Guo H., Liu M., Zhang H., Yang M. (2012). Pyrosequencing analysis of eukaryotic and bacterial communities in faucet biofilms. Sci. Total Environ..

[72] Chao Y., Mao Y., Wang Z., Zhang T. (2015). Diversity and functions of bacterial community in drinking water biofilms revealed by high-throughput sequencing. Sci. Rep..

[73] Madigan M., Martinko J. (2005). Brock Biology of Microorganisms.

[74] Carew M.E., Pettigrove V.J., Metzeling L., Hoffmann A.A. (2013). Environmental monitoring using nextgeneration sequencing: rapid identification of macroinvertebrate bioindicator lineages. Front. Zool..

[75] Kisan V., Valente A., Lahm A., Tanet G., Lettieri T. (2012). Phylogenetic and functional metagenomic profiling for assessing microbial biodiversity in environmental monitoring. PLoS One.

[76] Hajibabaei M., Shokralla S., Zhou X., Singer G.A., Baird D.J. (2011). Environmental barcoding: a nextgeneration sequencing approach for biomonitoring applications using river benthos. PLoS One.

[77] Logares R., Lindström E.S., Langenheder S., Logue J.B., Paterson H., Laybourn-Parry J., Rengefors K., Tranvik L., Bertilsson S. (2013). Biogeography of bacterial communities exposed to progressive long-term environmental change. ISME J..

